# To Dereplicate or Not To Dereplicate?

**DOI:** 10.1128/mSphere.00971-19

**Published:** 2020-05-20

**Authors:** Jacob T. Evans, Vincent J. Denef

**Affiliations:** a Department of Ecology and Evolutionary Biology, University of Michigan, Ann Arbor, Michigan, USA; University of Wisconsin—Madison

**Keywords:** MAG, binning, dereplication, metagenomics, population genomics, software

## Abstract

Metagenome-assembled genomes (MAGs) expand our understanding of microbial diversity, evolution, and ecology. Concerns have been raised on how sequencing, assembly, binning, and quality assessment tools may result in MAGs that do not reflect single populations in nature. Here, we reflect on another issue, i.e., how to handle highly similar MAGs assembled from independent data sets. Obtaining multiple genomic representatives for a species is highly valuable, as it allows for population genomic analyses; however, when retaining genomes of closely related populations, it complicates MAG quality assessment and abundance inferences. We show that (i) published data sets contain a large fraction of MAGs sharing >99% average nucleotide identity, (ii) different software packages and parameters used to resolve this redundancy remove very different numbers of MAGs, and (iii) the removal of closely related genomes leads to losses of population-specific auxiliary genes. Finally, we highlight some approaches that can infer strain-specific dynamics across a sample series without dereplication.

## Abstract (precis)

Metagenome-assembled genomes (MAGs) expand our understanding of microbial diversity, evolution, and ecology. Concerns have been raised on how sequencing, assembly, binning, and quality assessment tools may result in MAGs that do not reflect single populations in nature. Here, we reflect on another issue, i.e., how to handle highly similar MAGs assembled from independent data sets. Obtaining multiple genomic representatives for a species is highly valuable, as it allows for population genomic analyses; however, when retaining genomes of closely related populations, it complicates MAG quality assessment and abundance inferences.

## OPINION/HYPOTHESIS

**FIG 1:**
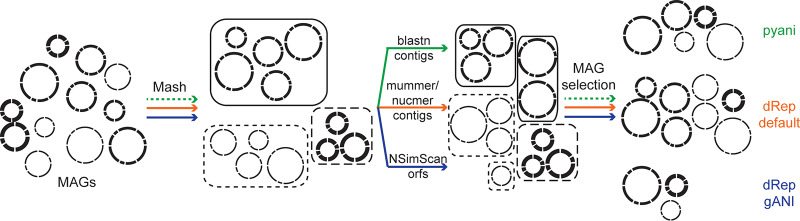
Overview of dereplication approaches used in this study. All approaches first cluster similar genomes (Mash clusters are delineated with boxes) using a fast, less accurate approach (Mash), which is included in the dRep package but is a separate preprocessing step we carried out for the pyani analysis (indicated with the dotted line). Each cluster of MAGs then is separately dereplicated using pairwise alignments by identifying MAGs within each Mash cluster that share ANI above the specified threshold. These clusters are indicated by boxes, with Mash clusters split in two multiple cluster groups using the same line type (full or dashed lines). Which genomes end up in the same cluster varies depending on the approach used; only one clustering is shown. Finally, a representative MAG is selected, either as part of the package (dRep) or using a custom script (our approach that used pyani for pairwise comparisons, indicated by the dotted line), selecting the MAG with the highest estimated completion and lowest estimated contamination.

While initially the reconstruction of metagenome-assembled genomes (MAGs) was only achievable in lower-diversity or highly uneven communities (1), in the past 5 years, reports on the reconstruction of hundreds to thousands of MAGs have become routine (2, 3). In the past year, highly automated assembly and binning pipelines have accelerated this trend (4, 5). These advances open up exciting prospects for addressing questions regarding the physiology, ecology, and evolution of microbial life. However, MAGs are inherently less reliable than isolate genomes due to their assembly and binning from DNA sequences originating from a mixed community. Various reports have highlighted issues associated with MAGs. Misassemblies and/or incorrect binning can lead to composite genomes that contain contigs originating from independent genomes, which can lead to incorrect inferences about the metabolic potential and phylogenetic novelty of populations represented by these MAGs (6, 7). Additionally, binning software performs poorly on contigs smaller than 2 to 5 kb (8); hence, smaller contigs generally are excluded from binning procedures. However, the assembly of metagenomic data from communities containing closely related strains often leads to highly fragmented assemblies with many contigs below these cutoffs, leading to incomplete genomes that can lead to wrong conclusions regarding ecological differentiation between populations (9). As sample series from the same environment across time or space typically will sample many closely related populations, the independent assembly of each sample is often preferable to avoid assembly fragmentation due to genomic variation between conspecific populations in different samples. However, this often leads to highly similar MAGs being generated across the sample data set. Therefore, multiple tools have been developed to remove these seemingly redundant MAGs, mainly based on average nucleotide identity (ANI) between MAGs after sequence alignment. Generally, due to the high number of MAGs, which makes doing all pairwise alignments too computationally intensive, MAGs are first grouped using a fast but less accurate alignment method, such as Mash (10). This step is included in the tool dRep, which subsequently uses genomewide alignments using animf with mummer/nucmer to align contigs (dRep default [11–13]) or gene-based alignments using NSimScan to align open reading frames identified in each MAG (dRep gANI [14]). Other tools, such as pyani (15), calculate sequence identity using BLAST-based genomewide alignments of MAG contigs. While slower, we consider it the reference for comparison due to the higher accuracy of BLAST-based alignments (16) (Fig. 1). When using Mash as a step preceding pyani, we used default parameters (10). The computed pairwise distances then were used to cluster genomes into similar groups with hierarchical clustering using a custom python script with fcluster from SciPy (http://www.scipy.org/) with a threshold of 2 (https://github.com/DenefLab/Dereplication-Letter-Code). pyani then was run within each group created from the clustering (Fig. 1). We used the recommended default percent sequence alignment threshold for dRep (10%) but used 75% for pyani, similar to the cutoff used for identifying orthologs using BLAST alignments. Only MAGs meeting this threshold with both comparisons from the pairwise comparison were dereplicated.

## WHY DEREPLICATE?

**FIG 2:**
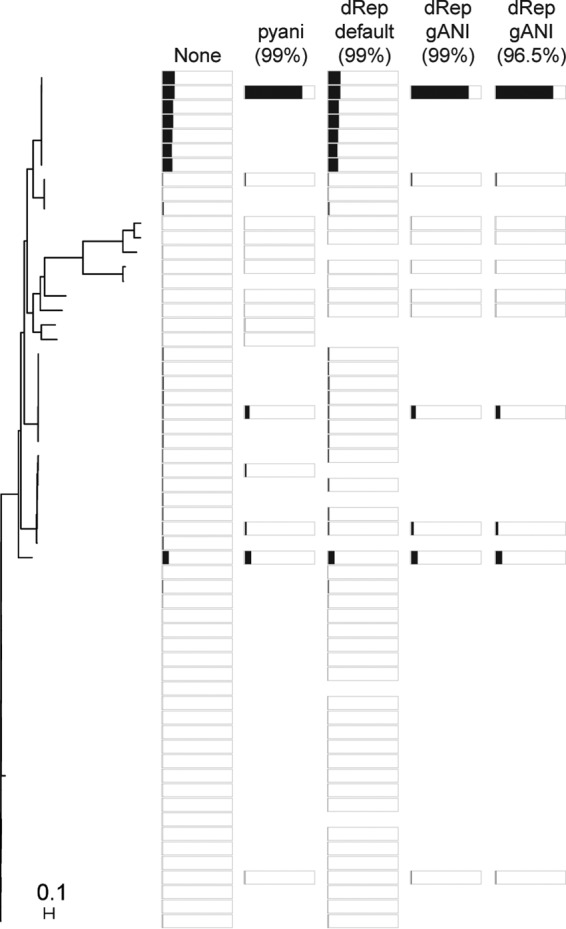
Effects of dereplication. Phylogenetic tree of a set of closely related MAGs (family *Muribaculaceae*) from Parks et al. (3), grouped based on sequence similarity by Mash. A box outline indicates the genome was preserved after dereplication, while white space indicates it was removed. The dRep default does not remove multiple nearly identical MAGs, while dRep-gANI removes MAGs that are more distantly related than the 99% or 96.5% ANI cutoff. Black bars show the average sequence read coverage across all contigs of each MAG, ranging from 0 to 2,000, when aligning a metagenomic data set (Sequence Read Archive accession no. SRR1702559) using all genomes in the tree (none) or dereplicated genome sets using different tools. Reads were mapped to each Multi-FASTA file of retained MAGs using BWA-MEM with default parameters (26). Average coverage per contig was computed with pileup.sh from bbtools (https://sourceforge.net/projects/bbmap/). The phylogenetic tree was created by searching for marker genes with PhyloSift (27) using its default set of marker genes. All MAGs had estimated completeness levels of >90% (3). The genes then were aligned with PhyloSift and the resulting alignments concatenated, and the tree was created with FastTree (28) using the -nt and -gtr parameters.

Dereplication is the reduction of a set of genomes, typically assembled from metagenomic data, based on high sequence similarity (e.g., >99% average nucleotide identity) between these genomes. When redundancy in a database of genomes is maintained, the subsequent step of mapping sequencing reads back to this database of genomes leads to sequencing reads having multiple high-quality alignments. Depending on the software used and parameters chosen, this leads to sequencing reads either being randomly distributed across the redundant genomes, with one random alignment reported from many possible options, or being reported at all redundant locations. When using these data to make inferences about the relative abundance and population dynamics across samples, relative abundance for each taxon represented by these redundant MAGs will look artificially low, and it will appear that multiple ecologically equivalent populations co-occur. Instead, the more likely conclusion should be that one abundant population exists across all samples (Fig. 2). This issue has been acknowledged in multiple studies, and authors have chosen various cutoffs to avoid this issue (e.g., >95% average nucleotide identity [4], >98% average nucleotide identity [2], >95% amino acid identity [17], and >99.5% amino acid identity [3]). In our experience, the presence of multiple closely related genomes can also complicate manually curating MAGs using, for example, Anvi’o (18). When multiple similar genomes are present, particularly when some of the closely related MAGs are less complete, these differential coverage patterns will be less reliable due to the distribution of sequencing reads across MAGs in some parts of the genome and not in others, generating divergent differential coverage patterns for contigs originating from the same population. This could lead to the removal of parts of the genome that do in fact belong.

## WHY NOT DEREPLICATE?

Traditional population genomic analyses rely on the availability of genome sequences of many conspecific isolates. Metagenomics-based population genomic analyses could enhance cultivation-dependent methods by obtaining sequences of multiple closely related, independently sampled populations (a population being defined as individuals of the same species occurring at the same time and place). While many methodological issues remain (see reference 19), MAGs of >99% ANI originating from multiple samples could be a valuable resource, which would be lost if we dereplicate our MAG data sets. Most approaches to dereplicate remove genomes based on the sequence identity of shared parts of the genome. As such, when removing genomes, in addition to data on single-nucleotide variation, we may lose information on variability in the auxiliary gene content among representatives from the same species. As an example, we analyzed the effect of dereplication on database auxiliary gene content using two of the most commonly used tools (dRep and redundancy removal based on pyani results). We used a set of 46 Microcystis aeruginosa MAGs that we previously generated with extensive manual curation (9). The ANI between pairs of these 46 genomes averages to 96.4%. Out of a total of 9,175 unique gene clusters across the 46 MAGs, dereplication led to the removal of up to 2,228 auxiliary gene clusters when using dRep gANI with a 96.5% cutoff (used for species delineation using genome sequences [14]) (Fig. 2). At the other extreme, using the dRep default, no genomes were removed from the MAG set; thus, no gene clusters were lost, while intermediate numbers of gene clusters were removed when using pyani (213) and dRep gANI (447) at 99% thresholds.

## VARIABLE PERFORMANCE OF COMMONLY USED SOFTWARE

As already indicated from the analysis shown in the first part of Table 1, different dereplication tools lead to different outcomes, even when using the same sequence identity cutoffs. Using publicly available MAG data sets (3–5), we evaluated the performance of two commonly used dereplication tools, dRep and pyani. For dRep, we used the default parameters, a cutoff of 99% (dRep default), and the gANI option, which aligns predicted open reading frames rather than the entire genomes, with cutoffs of 99% and 96.5% (dRep gANI). For pyani, we used a 99% ANI cutoff. As outlined in Fig. 1, to reduce computation time, all approaches first cluster MAGs by calculating pairwise distances using Mash.

**TABLE 1:** Summary of comparison of dereplication tools

Parameter	No. of gene clusters/MAGs retained by dereplication tool (% identity cutoff)				
	None	Pyani (99%)	dRep-default (99%)	dRep-gANI (99%)	dRep-gANI (96.5%)
Effect of dereplication on retained pangenome gene clusters^*a*^					
Microcystis aeruginosa	9,175	8,962	9,175	8,728	6,947
Effect of dereplication on number of retained MAGs^*b*^					
Parks et al. (3) (all)	7,800	5,236	6,288	4,047	3,357
Almeida et al. (4) (novel MAGs)	1,951	1,865	1,607	1,605	1,590
Pasolli et al. (5) (SGB12451)	50	8	40	1	1
Pasolli et al. (5) (SGB1437)	49	36	41	26	1

a Retained gene clusters of the Microcystis aeruginosa pangenome of 46 MAGs (9). A gene cluster consists of all genes across all genomes that had a minimum bit score of at least 0.5 when using the pangenome analysis workflow in Anvi’o (18). Retention of at least one representative in each gene cluster was evaluated when using different dereplication tools and ANI settings.

b Number of MAGs remaining after dereplication tools were run shows tool-dependent results of dereplication using data from three published data sets. SGB refers to numbered species-level clusters generated in analyses by Pasolli et al. (5).

First, we performed a comprehensive analysis of a set of 7,800 genomes generated from 1,550 public metagenomes (3). In this study, no dereplication was done for most analyses, except for building the tree represented in Fig. 2 in the study originally reporting these MAGs (3). For the latter analysis by Parks and coauthors, dereplication was performed by removing genomes with an amino acid identity (AAI) of ≥99.5%, as calculated using CompareM (https://github.com/dparks1134/CompareM), resulting in the removal of 27.5% of all MAGs. In our own analyses, relative to the pyani reference (32.9% removal), default dRep removed fewer genomes (19.3%), while the gANI dRep approach removed more MAGs (48.1% [99% ANI], 56.9% [96.5% ANI]) (Table 1). A closer look at one cluster of related MAGs indicated that dRep gANI regularly removed genomes that did not require removal, while dRep with default parameters did not remove a sufficient number of MAGs (Fig. 2).

For a recent study that generated more than 90,000 MAGs (4), we performed our comparative dereplication analysis on the 1,952 uncultured bacterial species that were identified and examined by the authors. These were MAGs not classified at the species level in current databases that had been dereplicated by removing less complete MAGs that shared an ANI of >95% across 60% of their sequence length. In this case, pyani removed four times fewer MAGs than the different implementations of dRep (Table 2). In contrast to our preceding analyses, dRep default removed more MAGs than pyani, potentially because the authors had already dereplicated their MAG set at 95% ANI. Finally, we analyzed two MAG groups, clustered at the species level (95% ANI) by the authors of a recent study generating more than 150,000 MAGs (5). In this case, dRep default again removed fewer MAGs than pyani, while dRep using gANI removed many more MAGs (Table 2). Various factors may explain the differences in outcome between dereplication approaches, including the faster but less accurate alignment and percent identity calculation methods used in dRep, the diverging percent alignment thresholds used, and, potentially, different implementations of Mash in dRep and in our combined Mash-pyani approach. As for the percent alignment thresholds used, when we tried different coverage thresholds with the set of genomes represented in Fig. 2, dropping the coverage threshold for pyani to 10%, used by dRep, led to the removal of one extra MAG. When doing the same for the *Microcystis* MAG set, we observed no change in the number of genomes removed by pyani (10 to 75% alignment thresholds when using 100% complete MAGs) (Table 2).

**TABLE 2:** Impact of genome completeness and percent alignment threshold on dereplication using pyani

% alignment	Genome completeness^*a*^ (%)									
	10	20	30	40	50	60	70	80	90	100
10	37	35	36	33	33	31	29	27	23	21
25	45	44	37	33	33	31	29	27	23	21
50	45	45	45	45	40	31	29	27	23	21
75	45	45	45	45	45	45	44	38	24	21

a Microcystis aeruginosa MAGs were artificially reduced in completeness by random subsampling of contigs. The full MAGs ranged in estimated completeness between 95 and 100%. Shown are the numbers of the original 46 MAGs retained by our combined Mash-pyani approach using different percent alignment thresholds.

Finally, dereplication tools also vary in their performance based on the completeness of the MAGs that are being compared. Previous guidelines for the use of dRep have shown that Mash, the first step in all the tested dereplication approaches (Fig. 1), underestimates genome similarity for incomplete genomes and recommends against dereplicating genomes below 50% completeness (https://drep.readthedocs.io/en/latest/choosing_parameters.html). When using the pyani approach we favor, using our data set of *Microcystis* MAGs artificially reduced to lower completeness by randomly removing contigs, only 1 out of 46 genomes was removed at completeness levels between 10 and 60%. At 70, 80, and 90% completeness, pyani removed 2, 8, and 22 genomes at a threshold of >99% identity and alignments covering >75% of the MAG (Table 2). At their highest estimated completeness level, which ranged between 95 and 100%, 25 genomes were removed. While performance will likely vary depending on the data set, these results suggest that dereplication with our preferred combined Mash-pyani approach is not able to prune highly incomplete genomes and will retain more genomes than optimal even for genomes that are estimated to be 80 to 90% complete. Considering the limited effect of percent alignment threshold on the pyani approach when using complete MAGs, lowering this threshold helps remove more genomes and, hence, should be lowered to 50% or less when trying to prune more incomplete MAGs (Table 2).

## AVAILABLE APPROACHES TO LEVERAGE SAMPLING OF BETWEEN-POPULATION VARIATION

Several tools have been developed to maintain the auxiliary genomes of closely related strains while avoiding redundancy when tracking strain-resolved population dynamics in the environment using metagenomic data (reviewed in reference 19). They typically use metagenomic data in combination with a database of genomes of closely related isolates or MAGs based on whether alleles of shared genes (StrainPhlAn [20] and ConStrains [21]), strain-specific auxiliary genes (PanPhlAn [22]), or both are present in a sample (MIDAS [23]). Similarly, the Anvi’o package incorporates a metapangenome workflow that reduces a set of user-defined conspecific genomes to gene clusters representing core and auxiliary genes and then estimates strain abundances across metagenomic data sets (18). In principle, all of these approaches avoid the issues associated with database redundancy highlighted in Table 1 and Fig. 2 and the loss of population-specific auxiliary genes highlighted in Table 1. Although variant identification errors do remain, which are tool and likely database and metagenomic data set dependent, this has been reported to be as low as 0.1% (20). While potential issues with these approaches have not been fully evaluated, analyses focusing on populations where the dominant strain can be more readily resolved have been able to go as far as tracking *in situ* bacterial evolution in environmental biofilms and the human gut (24, 25).

## CONCLUSIONS

Genome-centric metagenomics has opened a view into the undescribed branches of the tree of life. However, full awareness of the risks associated with MAGs is needed to avoid the misinterpretation of the data and populating databases with questionable genomes. Dereplication is a step carried out by many researchers as part of metagenomic informatic pipelines, but we highlight large differences between commonly used tools in how many genomes are removed. Tools designed to resolve closely related genomes in a database exist and may circumvent issues with redundancy while maximally leveraging all data from conspecific populations obtained from generating MAGs. As the ability to resolve closely related genomes is dependent on the genetic distance between genomes in the database and between database genomes and those of sampled populations, these tools need broader adaptation and evaluation to fully evaluate their accuracy. Additionally, we hope there will be further development of tools that resolve the issues highlighted in this study.

## DATA AVAILABILITY

All code written and used for the analyses described in the manuscript can be found at https://github.com/DenefLab/Dereplication-Letter-Code.

## References

[1] Tyson GW, Chapman J, Hugenholtz P, Allen EE, Ram RJ, Richardson PM, Solovyev VV, Rubin EM, Rokhsar DS, Banfield JF 2004 Community structure and metabolism through reconstruction of microbial genomes from the environment. Nature 428:37–43. doi:10.1038/nature02340. 14961025

[2] Anantharaman K, Brown CT, Hug LA, Sharon I, Castelle CJ, Probst AJ, Thomas BC, Singh A, Wilkins MJ, Karaoz U, Brodie EL, Williams KH, Hubbard SS, Banfield JF 2016 Thousands of microbial genomes shed light on interconnected biogeochemical processes in an aquifer system. Nat Commun 7:13219. doi:10.1038/ncomms13219. 27774985 PMC5079060

[3] Parks DH, Rinke C, Chuvochina M, Chaumeil P-A, Woodcroft BJ, Evans PN, Hugenholtz P, Tyson GW 2017 Recovery of nearly 8,000 metagenome-assembled genomes substantially expands the tree of life. Nat Microbiol 2:1533–1542. doi:10.1038/s41564-017-0012-7. 28894102

[4] Almeida A, Mitchell AL, Boland M, Forster SC, Gloor GB, Tarkowska A, Lawley TD, Finn RD 2019 A new genomic blueprint of the human gut microbiota. Nature 568:499–504. doi:10.1038/s41586-019-0965-1. 30745586 PMC6784870

[5] Pasolli E, Asnicar F, Manara S, Zolfo M, Karcher N, Armanini F, Beghini F, Manghi P, Tett A, Ghensi P, Collado MC, Rice BL, DuLong C, Morgan XC, Golden CD, Quince C, Huttenhower C, Segata N 2019 Extensive unexplored human microbiome diversity revealed by over 150,000 genomes from metagenomes spanning age, geography, and lifestyle. Cell 176:649–662. doi:10.1016/j.cell.2019.01.001. 30661755 PMC6349461

[6] Shaiber A, Eren AM 2019 Composite metagenome-assembled genomes reduce the quality of public genome repositories. mBio 10:e00725-19. doi:10.1128/mBio.00725-19. 31164461 PMC6550520

[7] Chen L-X, Anantharaman K, Shaiber A, Eren AM, Banfield JF 2020 Accurate and complete genomes from metagenomes. Genome Res 30:315–333. doi:10.1101/gr.258640.119. 32188701 PMC7111523

[8] Dick G, Andersson A, Baker B, Simmons S, Thomas B, Yelton AP, Banfield J 2009 Community-wide analysis of microbial genome sequence signatures. Genome Biol 10:R85. doi:10.1186/gb-2009-10-8-r85. 19698104 PMC2745766

[9] Jackrel SL, White JD, Evans JT, Buffin K, Hayden K, Sarnelle O, Denef VJ 2019 Genome evolution and host-microbiome shifts correspond with intraspecific niche divergence within harmful algal bloom-forming Microcystis aeruginosa. Mol Ecol 28:3994–4011. doi:10.1111/mec.15198. 31344288

[10] Ondov BD, Treangen TJ, Melsted P, Mallonee AB, Bergman NH, Koren S, Phillippy AM 2016 Mash: fast genome and metagenome distance estimation using MinHash. Genome Biol 17:132. doi:10.1186/s13059-016-0997-x. 27323842 PMC4915045

[11] Richter M, Rosselló-Móra R 2009 Shifting the genomic gold standard for the prokaryotic species definition. Proc Natl Acad Sci U S A 106:19126–19131. doi:10.1073/pnas.0906412106. 19855009 PMC2776425

[12] Olm MR, Brown CT, Brooks B, Banfield JF 2017 dRep: a tool for fast and accurate genomic comparisons that enables improved genome recovery from metagenomes through de-replication. ISME J 11:2864–2868. doi:10.1038/ismej.2017.126. 28742071 PMC5702732

[13] Kurtz S, Phillippy A, Delcher AL, Smoot M, Shumway M, Antonescu C, Salzberg SL 2004 Versatile and open software for comparing large genomes. Genome Biol 5:R12. doi:10.1186/gb-2004-5-2-r12. 14759262 PMC395750

[14] Varghese NJ, Mukherjee S, Ivanova N, Konstantinidis KT, Mavrommatis K, Kyrpides NC, Pati A 2015 Microbial species delineation using whole genome sequences. Nucleic Acids Res 43:6761–6771. doi:10.1093/nar/gkv657. 26150420 PMC4538840

[15] Pritchard L, Glover RH, Humphris S, Elphinstone JG, Toth IK 2016 Genomics and taxonomy in diagnostics for food security: soft-rotting enterobacterial plant pathogens. Anal Methods 8:12–24. doi:10.1039/C5AY02550H.

[16] Yoon S-H, Ha S-M, Lim J, Kwon S, Chun J 2017 A large-scale evaluation of algorithms to calculate average nucleotide identity. Antonie Van Leeuwenhoek 110:1281–1286. doi:10.1007/s10482-017-0844-4. 28204908

[17] Woodcroft BJ, Singleton CM, Boyd JA, Evans PN, Emerson JB, Zayed AAF, Hoelzle RD, Lamberton TO, McCalley CK, Hodgkins SB, Wilson RM, Purvine SO, Nicora CD, Li C, Frolking S, Chanton JP, Crill PM, Saleska SR, Rich VI, Tyson GW 2018 Genome-centric view of carbon processing in thawing permafrost. Nature 560:49–54. doi:10.1038/s41586-018-0338-1. 30013118

[18] Delmont TO, Eren AM 2018 Linking pangenomes and metagenomes: the Prochlorococcus metapangenome. PeerJ 6:e4320. doi:10.7717/peerj.4320. 29423345 PMC5804319

[19] Denef VJ 2018 Peering into the genetic makeup of natural microbial populations using metagenomics, p 49–75. *In* Polz M, Rajora OP (ed), Population genomics: microorganisms. Springer International Publishing, New York, NY.

[20] Truong DT, Tett A, Pasolli E, Huttenhower C, Segata N 2017 Microbial strain-level population structure and genetic diversity from metagenomes. Genome Res 27:626–638. doi:10.1101/gr.216242.116. 28167665 PMC5378180

[21] Luo C, Knight R, Siljander H, Knip M, Xavier RJ, Gevers D 2015 ConStrains identifies microbial strains in metagenomic datasets. Nat Biotechnol 33:1045–1052. doi:10.1038/nbt.3319. 26344404 PMC4676274

[22] Scholz M, Ward DV, Pasolli E, Tolio T, Zolfo M, Asnicar F, Truong DT, Tett A, Morrow AL, Segata N 2016 Strain-level microbial epidemiology and population genomics from shotgun metagenomics. Nat Methods 13:435–438. doi:10.1038/nmeth.3802. 26999001

[23] Nayfach S, Rodriguez-Mueller B, Garud N, Pollard KS 2016 An integrated metagenomics pipeline for strain profiling reveals novel patterns of bacterial transmission and biogeography. Genome Res 26:1612–1625. doi:10.1101/gr.201863.115. 27803195 PMC5088602

[24] Denef VJ, Banfield JF 2012 In situ evolutionary rate measurements show ecological success of recently emerged bacterial hybrids. Science 336:462–466. doi:10.1126/science.1218389. 22539719

[25] Garud NR, Good BH, Hallatschek O, Pollard KS 2019 Evolutionary dynamics of bacteria in the gut microbiome within and across hosts. PLoS Biol 17:e3000102. doi:10.1371/journal.pbio.3000102. 30673701 PMC6361464

[26] Li H 2013 Aligning sequence reads, clone sequences and assembly contigs with BWA-MEM. arXiv 1303.3997 [q-bio.GN] https://arxiv.org/abs/1303.3997.

[27] Darling AE, Jospin G, Lowe E, Matsen FA IV, Bik HM, Eisen JA 2014 PhyloSift: phylogenetic analysis of genomes and metagenomes. PeerJ 2:e243. doi:10.7717/peerj.243. 24482762 PMC3897386

[28] Price MN, Dehal PS, Arkin AP 2009 FastTree: computing large minimum evolution trees with profiles instead of a distance matrix. Mol Biol Evol 26:1641–1650. doi:10.1093/molbev/msp077. 19377059 PMC2693737

